# The psychological reality of the learned “*p* < .05” boundary

**DOI:** 10.1186/s41235-024-00553-x

**Published:** 2024-05-03

**Authors:** V. N. Vimal Rao, Jeffrey K. Bye, Sashank Varma

**Affiliations:** 1 Department of Educational Psychology, University of Minnesota, 56 E River Road Room 250, Minneapolis, MN 55455 USA; 2 School of Interactive Computing and School of Psychology, Technology Square Research Building, Georgia Institute of Technology, 85 5Th St NW, Atlanta, GA 30308 USA

**Keywords:** *p*-values, Statistical thinking, Categorical perception, Meta-science, Replication crisis

## Abstract

The .05 boundary within Null Hypothesis Statistical Testing (NHST) “has made a lot of people very angry and been widely regarded as a bad move” (to quote Douglas Adams). Here, we move past meta-scientific arguments and ask an empirical question: What is the psychological standing of the .05 boundary for statistical significance? We find that graduate students in the psychological sciences show a *boundary effect* when relating *p*-values across .05. We propose this psychological boundary is learned through statistical training in NHST and reading a scientific literature replete with *“statistical significance”*. Consistent with this proposal, undergraduates do not show the same sensitivity to the .05 boundary. Additionally, the size of a graduate student’s boundary effect is not associated with their explicit endorsement of questionable research practices. These findings suggest that training creates distortions in initial processing of *p*-values, but these might be dampened through scientific processes operating over longer timescales.

**Supplementary Information:**

The online version contains supplementary material available at 10.1186/s41235-024-00553-x.

## Significance statement

Null Hypothesis Statistical Testing (NHST) uses *p*-values to quantify the consistency between observed evidence and the predictions of scientific hypotheses. By arbitrary convention, psychological scientists adopt .05 as the boundary between hypotheses that are “statistically significant” and those that are not. The pressure to achieve “significant” results may be one reason why researchers engage in questionable research practices, and one cause for the replication crisis more generally. We investigated whether through statistical training and reading a scientific literature still dominated by NHST, .05 becomes a psychological boundary in the minds of emerging psychological scientists. This was the case. Our findings raise the meta-science question of how the distortions in initial processing of *p*-values demonstrated here are dampened through the long-term processes of science. They also suggest that competitors to NHST that also include “magic numbers” may be susceptible to the same problems brought by the .05 boundary.

## Introduction

The long history of calls against Null Hypothesis Significance Testing (NHST) has accelerated over the past 15 years (e.g., Cumming, 2014; Kruschke & Liddell, 2018). A central villain in this story has been the 0.05 boundary that by convention demarcates “statistically significant” hypotheses from their null brethren (Wasserstein et al., 2019). Fall on the “virtuous” side of this boundary and take the first step towards publication and scientific glory (Simonsohn et al., 2014). Fall on the wrong side and find yourself on the path to abandoned projects and file-drawered manuscripts (Rosenthal, 1979).

Within NHST, the 0.05 boundary has been criticized as partially to blame for the replication crisis (Benjamin et al., 2018; Ioannidis, 2005; Simonsohn et al., 2014). This value may be too high, resulting in a scientific literature filled with too many false positives (Ioannidis, 2005). For this reason, some have argued for shifting the boundary downward, for example to the more conservative 0.005 boundary (Benjamin et al., 2018). More insidious is the possibility that the presence of *any* boundary may cause scientists to distort their behavior to push their results to the “virtuous” side (Neuliep & Crandall, 1993; Rosenthal, 1979), for example by engaging in Questionable Research Practices (QRPs; John et al., 2012). In the quest to reduce costs and achieve the ever-increasing publication rates necessary for academic success, scientists are incentivized to run studies with small sample sizes, which have a higher probability of producing false positives with QRPs such as optional stopping (Simmons et al., 2011) and selective reporting (Ioannidis & Trikalinos, 2007). More broadly, the culture of science might discourage scientists from replicating findings that are “already known” (Neuliep & Crandall, 1993)—and when scientists attempt such replications and “fail”, they may be discouraged from publishing by journals uninterested in “null results” (Rosenthal, 1979).

These problems have caused some to call for moving away from NHST and the 0.05 boundary. One alternative emphasizes effect sizes and interpretations of their “practical” significance rather than statistical significance (Cumming, 2014). Another alternative promotes the use of Bayesian statistics, claimed to be more consistent with human intuitions about the nature of knowledge and evidence evaluation (Kruschke & Liddell, 2018).

Here, we ask a different question, one that is not normative but empirical in nature: What is the *psychological* standing of the 0.05 boundary for statistical significance? (See Bishop, 2020 for other empirical questions about the psychology of research practice.) We teach psychology graduate students about 0.05 as a matter of convention. At the same time, they read heavy doses of a scientific literature where results have long been cast in terms of the “*p* < 0.05” boundary. Our central claim is that through this training, emerging scientists come to internalize 0.05 as a psychological boundary between two categories that often affect publication: results that are “statistically significant” and those that are not. Subsequently, this boundary distorts their processing of *p*-values in the talks they hear, the papers they read, and the statistical results they generate in their own research. Thus, we predict that a *boundary effect*–akin to a categorical perception effect (CPE)^1^–follows from this training, such that pairs of *p*-values that straddle 0.05 will be processed as relatively different, whereas equidistant pairs on the same side of 0.05 will be processed as relatively similar.

We are not the first to suggest that 0.05 might be a psychological boundary for scientists. Prior research on researchers’ *confidence* in the presence of an effect as a function of a test’s *p*-value suggests discontinuities at *p* = 0.05 (Beauchamp & May, 1964; Nelson et al., 1986; Rosenthal & Gaito, 1963) and other nonlinearities across the range of *p*-values (Poitevineau & LeCoutre, 2001). More recently, researchers have investigated the role that confidence intervals play in these discontinuities (e.g., Coulson et al., 2010; Helske et al., 2021; Hoekstra et al., 2012). Our research focuses instead on lower-level speeded judgments of the *similarity* of *p*-values outside of the context of confidence in a particular result, while carefully controlling for mathematical cognition effects (Ashcraft, 1992). This situates our experiment in the emerging focus on statistical cognition (e.g., Ciccione & Dehaene, 2021).

**Fig. 1:**
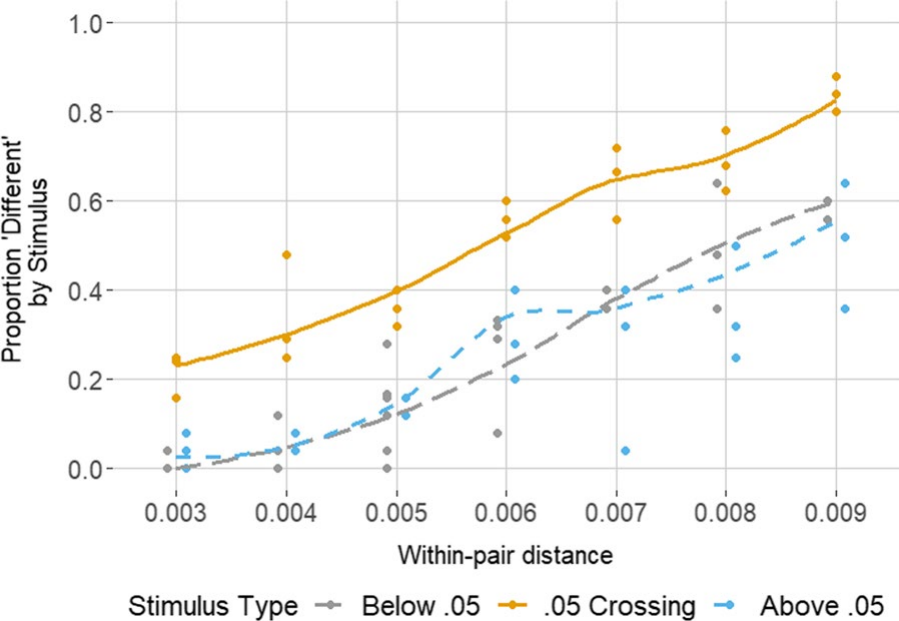
Results of Rao et al. (2022). On average, for each unique stimulus (each dot) across the range of within-pair distances, graduate students were more likely to judge two p-values as ‘different’ when they crossed the 0.05 boundary (solid orange line) than when they crossed a different hundredths boundary, i.e., one above (short-dashed blue line) or below (long-dashed grey line)

Recently, we investigated whether graduate students in the psychological sciences show a boundary effect at 0.05 when judging the similarity of pairs of *p*-values by adapting the AX paradigm from the categorical perception literature (Rao et al., 2022). Participants saw two *p*-values and made speeded judgments of whether they were “different” or “similar”. They were more likely to judge *p*-values as “different” when they crossed 0.05 (e.g., {0.043, 0.057}) versus when they crossed another nearby hundredths boundary (e.g., {0.023, 0.037} or {0.063, 0.077}). This held regardless of the arithmetic distance between the *p*-values (see Fig. 1).

The current study goes beyond prior work in three important ways. First, we previously proposed that the psychological boundary for graduate students was due to their statistical training in NHST and the literature they read (Rao et al., 2022). However, an alternate explanation for the results is that 0.05 is a boundary for *all* people. Mathematical cognition research has shown that some numbers are landmarks on the mental number line (MNL). For example, the 10s are psychological boundaries for people whose culture expresses numbers using base-10 notation (Nuerk et al., 2011). Furthermore, when reasoning about fractions, people use ½ as a landmark (Barth & Paladino, 2011). It is therefore possible that when reasoning about rational numbers (including *p*-values) expressed as proportions or probabilities, people use 0.5 and 0.05 as *domain-general* landmarks. To rule out the possibility that 0.05 is simply a psychological boundary for everyone, we replicated our prior experiment (Rao et al., 2022) while adding a comparison group of statistically novice undergraduate students. We predicted that emerging psychological scientists would show a boundary effect above and beyond any domain-general landmark effect when judging the similarity (or difference) of two *p*-values across 0.05.

Second, the AX paradigm on its own presents relatively weak evidence for the existence of CPE-like psychological boundaries. Thus, we added the stronger ABX paradigm (e.g., Greenaway, 2017; see Repp, 1984, for more information about the ABX paradigm). In this paradigm, participants see a triad of values from a continuous scale (typically perceptual) and must judge whether the value on the left or right end is more like the middle value. We had participants make this judgment for triads of *p*-values such as {0.043, 0.049, 0.054}. Here, the arithmetically correct answer is 0.054 because $\left|.049-.054\right|<\left|.043-.049\right|$. However, we propose that emerging scientists experience a CPE-like boundary at 0.05, and this increases the *psychological* distance between 0.049 and 0.054 while decreasing the *psychological* distance between 0.043 and 0.049. Therefore, we predict they will be less likely to choose the numerically closer end value, with the comparison group again being undergraduates.

Since undergraduates have not received this domain-specific training around 0.05, we predict they will experience no special *p*-value boundary at 0.05; instead, their judgments should be driven by domain-general landmarks (e.g., hundredths-place values) and their intuitive sense of numerical distance, following the predictions of the logarithmically-compressed MNL model that is supported by decades of research in numerical cognition (Dehaene, 2003; Moyer & Landauer, 1967).

Third, we investigated whether there is an association between the initial processing of *p*-values and explicit beliefs about QRPs. We adapted the 10-item survey developed by John et al. (2012) to measure emerging scientists’ explicit beliefs about the acceptability of various QRPs (e.g., optional stopping). We compared these beliefs to quantifications of their individual boundary effects for 0.05 in the AX and ABX tasks. We hypothesized that individuals who experience a larger psychological boundary at 0.05 may also find QRPs more acceptable. This is important for potentially linking the moment-by-moment cognitive processing of emerging scientists to the replication crisis in psychological science more generally.

## Materials and methods

### Power analysis

Our previous study (Rao et al., 2022) estimated the median rate ratio at which graduate students judge *p*-values that cross 0.05 as “different” to be 1.96 times the rate for *p*-values that are both on the same side of 0.05. A goal of the current study was to be able to detect a medium effect by computing the relative difference in the rate ratios between undergraduate and graduate students. We expected undergraduate students to also show a boundary effect due to the special significance of 0.05 as a general landmark in numerical processing, though one smaller than that of graduate students. We therefore predicted there would be an average rate ratio of 1.4 (the square root of 1.96) for undergraduates with a standard deviation of 0.5 for the logarithm of rate ratios between undergraduates and graduate students (based on the results of Rao et al., 2022). Given these parameters and a desire to detect a relative difference in ratios between the two groups of approximately 1.4 with 80% power and 5% Type I error rate, we estimated that we needed to recruit at least 34 participants per group. Because of the novelty of using the ABX task with numerical stimuli, we deliberately oversampled both groups.

### Participants

To study emerging scientists in the psychological sciences, we recruited graduate students in the psychological sciences from a large research university in the Midwestern US. We chose the sample size based on prior results and the power analysis detailed above.

A total of 42 graduate students completed the study (see Table 1 for demographics and Additional file 1: Table S1 for information about their areas of specialization). They were recruited via email, had previously completed a yearlong sequence in statistical methods at the doctoral level, and were in at least their second full year of graduate studies. A total of 49 undergraduate students completed the study (see Table 1 for demographics and Additional file 1: Table S2 for information about their major disciplines). They were recruited from the same university via email, had previously taken an introductory course in educational psychology, and had participated in prior studies in our lab. Data for some participants in each group were excluded from some of the analyses for failure to comply with task instructions, as described in the Results.

**Table 1:** Gender and race/ethnicity identity for study participants by group

Attribute	Participant group	
	Graduate students	Undergraduate students
Total	42	49
Gender identity		
Man	11	10
Woman	29	36
Non-binary	1	2
Chose not to respond	1	1
Race/ethnicity (select all that apply)		
Alaska Native or American Indian	0	0
Asian	17	15
Black or African American	0	0
Hispanic, Latino, or Spanish Origin	1	0
Middle Eastern or North African	1	1
Pacific Islander	0	0
White	24	35
Other	0	0
Chose not to respond	0	0

#### Constraints on generality

This study’s population of interest is emerging scientists in the psychological sciences. We define them as graduate students with between 1 and 5 years of experience conducting psychological research, and with sufficient statistical training to understand *p*-values. Our sample frame included all current graduate students in the psychological sciences in a large research university in the Midwestern US who had completed at least one year of graduate-level statistics. Although the study information was emailed to all eligible graduate students, those who elected to participate are not a fully representative sample of their department demographics, much less demographically representative of all emerging scientists in the psychological sciences across the US, let alone worldwide. However, the theoretical mechanisms causing a possible boundary effect should not differentially affect members of the target population based on any demographic characteristics. Other factors such as statistics curricula, prior mathematics and statistical experiences, math anxiety, and prevalence of statistical tests in participants’ sub-fields may all be related to participants’ selection in the study tasks. This study did not explicitly control for these macro factors.

This study also used a control group of statistically untrained undergraduate students, defined as undergraduate students who have not yet completed a post-secondary statistics course. Our sample frame included undergraduate students who had previously completed a course in educational psychology at a large research university in the Midwestern US or had completed a study in our lab in the past, having been recruited either through flyers, social media, or word of mouth. Thus, this sample is not representative of all undergraduates at the university, much less all statistically untrained undergraduates across the US or worldwide. Once again, the theoretical mechanisms causing a possible boundary effect should not differentially affect members of the target population based on any demographic characteristics. Future studies may recruit from a broader participant pool for both groups in order to verify this study’s findings across academic institutions and cultures.

### Materials

The graduate student participants completed four tasks in the following order: the AX discrimination task, an unrelated filler task, the ABX discrimination task, and a survey assessing their beliefs about QRPs. Undergraduate participants only completed the AX and ABX tasks because the filler task was for a separate study for which undergraduate participants were not in the eligible participant pool. The goal of the AX and ABX tasks was to find converging evidence for the hypothesis that statistical training in the psychological sciences results in a CPE-like boundary at 0.05. The critical contrast is whether participants’ responses differ when the values cross 0.05, relative to when they cross hundredths boundaries above or below 0.05 (e.g., 0.07 or 0.03, respectively), and whether this relative difference is exaggerated for graduate students relative to undergraduates.

#### The AX task

In this task, participants were provided with two *p*-values and asked to judge whether they were “similar” or “different”. Note that there is no objectively correct answer for such judgments. There were 150 stimulus pairs. Fifty experimental pairs crossed 0.05 (“0.05 Crossing”, e.g., 0.043 vs. 0.057). Within this set, the distance between the two *p*-values was systematically varied between 0.002 and 0.014; see Table 2 for example stimuli. This enabled evaluation of whether the predicted effect of crossing the 0.05 boundary varied as a function of distance, a factor known to affect number comparison (Moyer & Landauer, 1967).

**Table 2:** Example stimuli for the AX task by stimulus type and within-pair distance

Distance	Stimulus Type		
	Below	0.05 Crossing	Above
0.002	{0.019, 0.021}	{0.049, 0.051}	{0.069, 0.071}
0.003	{0.038, 0.041}	{0.048, 0.051}	{0.058, 0.061}
0.004	{0.028, 0.032}	{0.048, 0.052}	{0.078, 0.082}
0.005	{0.027, 0.032}	{0.047, 0.052}	{0.057, 0.062}
0.006	{0.017, 0.023}	{0.047, 0.053}	{0.067, 0.073}
0.007	{0.039, 0.046}	{0.049, 0.056}	{0.079, 0.086}
0.008	{0.026, 0.034}	{0.046, 0.054}	{0.076, 0.084}
0.009	{0.018, 0.027}	{0.048, 0.057}	{0.068, 0.077}
0.011	{0.034, 0.045}	{0.044, 0.055}	{0.054, 0.065}
0.012	{0.024, 0.036}	{0.044, 0.056}	{0.064, 0.076}
0.013	{0.031, 0.044}	{0.041, 0.054}	{0.071, 0.086}
0.014	{0.013, 0.027}	{0.043, 0.057}	{0.053, 0.067}

The remaining stimulus pairs formed the control pairs. For these, the *p*-values were either both below 0.05 or both above 0.05, and also crossed a hundredths boundary. Their thousandths digits were yoked to those of the experimental pairs. For example, the experimental pair {0.043, 0.057} generated one Below 0.05 control pair {0.023, 0.037} and one Above 0.05 control pair {0.063, 0.077}. We constructed the control pairs in this way to balance the size effect, which also affects number comparison (Parkman, 1971). This is the finding that smaller numbers (e.g., {0.023, 0.037}) are perceived as farther apart than larger numbers (e.g., {0.063, 0.077}), consistent with a MNL representation that is log-compressed (Varma & Karl, 2013). Thus, we include the numerical magnitude (i.e., the size) of each stimulus as a covariate in our statistical model, and after having equated for the size effect in this manner, we compare the experimental pair that crosses 0.05 (e.g., {0.043, 0.057}) to the average of two yoked pairs, one below 0.05 and one above 0.05. This approach enables us to measure whether a boundary effect at 0.05 exists above and beyond any boundary effects at other hundredths-place boundaries like 0.03 and 0.07, after adjusting for the log-compressed MNL.

Critically, all three pairs had zero in the tenths place, the same digits in the thousandths place, and the same arithmetic distance between *p*-values. These constraints enabled us to control for the distance and decade-crossing effects (Nuerk et al., 2011; Schneider et al., 2020) found in mathematical cognition research when comparing the experimental vs. control pairs. Including control pairs both above and below 0.05 additionally controlled for the size effect (Parkman, 1971) as described above.

#### The ABX task

In this task, each stimulus consisted of an ordered triad of *p*-values, and participants had to judge which of the end values the middle value was “more like”. In the conventional version of this task, the three values are perceptual in nature and equidistant from each other along some physical continuum, for example different food products with varying amounts of salt to measure participants’ perceptions of taste (e.g., Greenaway, 2017). In these traditional perceptual experiments, that the three values are equidistant makes the “more like” and “same” judgments nonsensical to the extent that people have direct access to the raw physical sensations. However, the noise inherent in perceptual processing means that in practice, people are willing to make such judgments. The current study used numerical stimuli, not perceptual stimuli. It included *symmetric triads* where the middle *p*-value was equidistant from the two end *p*-values, e.g., {0.048, 0.051, 0.054}. If 0.05 is a psychological boundary, participants should be more likely to judge the end *p*-value on the same side of the boundary (e.g., 0.054) as “more like” the middle *p*-value (e.g., 0.051). However, there is not necessarily the same noise in processing numerical values as there is in processing perceptual values, especially given the ability to exactly compute the arithmetic differences via subtraction. Not surprisingly, then, some pilot participants commented on the infelicity of the symmetric triads, and so we treated them as filler trials in the present experiment.

Critically, we extended the ABX task to also include *asymmetric triads*, where the end *p*-value on the same side of 0.05 is also arithmetically further from the middle *p*-value, e.g., {0.043, 0.049, 054}, where $\left|.043-.049\right|>\left|.054-.049\right|$. For these triads, the arithmetically correct answer is the *p*-value across 0.05 from the middle value. If graduate students’ judgments are driven by a domain-specific boundary effect, then they should be less likely to make the arithmetically correct choice than undergraduate students. We adopted the “more like” phrasing to encourage judgments from intuition, especially given the speeded nature of the task. There were 102 asymmetric triads. Of these, 34 were experimental triads where one of the end *p*-values crossed 0.05 (e.g., {0.042, 0.048, 0.052}, {0.045, 0.051, 0.059}). The distance between the two *p*-values on the same side of the boundary varied between 0.003 and 0.008. The distance between the middle *p*-value and the *p*-value on the opposite side of the boundary varied between 0.002 and 0.007, and critically, this was always at least 0.001 less than the distance between the two *p*-values on the same side of the boundary. Each experimental triad was used to generate control triads in the same manner as for the AX task, with one spanning a hundredths barrier Below 0.05 (e.g., {0.025, 0.031, 0.039}) and the other one Above 0.05 (e.g., {0.065, 0.071, 0.079}). Again, this construction process ensured that the stimuli controlled for the well-known distance, decade crossing, and size effects in the mathematical cognition literature (Ashcraft, 1992). See Table 3 for example asymmetric triads.

**Table 3:** Example stimuli for the Asymmetric ABX task by stimulus type and within-triad distance

Hundredths crossing distance	Stimulus type		
	Below	0.05 Crossing	Above
0.002	{0.029, 0.031, 0.034}	{0.049, 0.051, 0.054}	{0.059, 0.061, 0.064}
0.003	{0.015, 0.019, 0.022}	{0.045, 0.049, 0.052}	{0.055, 0.059, 0.062}
0.004	{0.028, 0.032, 0.038}	{0.048, 0.052, 0.058}	{0.068, 0.072, 0.078}
0.005	{0.023, 0.029, 0.034}	{0.043, 0.049, 0.054}	{0.073, 0.079, 0.084}
0.006	{0.016, 0.022, 0.029}	{0.046, 0.052, 0.059}	{0.056, 0.062, 0.069}
0.007	{0.021, 0.029, 0.036}	{0.041, 0.049, 0.056}	{0.061, 0.069, 0.076}

#### The QRP survey

All graduate students completed a short survey about their beliefs related to QRPs. (Undergraduate participants did not receive this survey because they were unlikely to have had previous research experience involving making such decisions.) Participants were asked to respond to 10 items providing examples of QRPs and to rate “how often they are acceptable”. The items were those used by John et al. (2012), with the exception of item (5).^2^ That study recruited full-time faculty with experience writing and reviewing peer-reviewed publications, and asked them to rate how “defensible” each QRP was. Because our participants were less likely to have had such experience, we instead used the word “acceptable”, to reflect that their responses were more driven by the training they were receiving in their coursework and research labs. See Table 4 for the items measuring QRPs.

**Table 4:** Survey items measuring participants’ relative frequencies of the acceptability of questionable research practices, adapted from John et al. (2012); our implementation of the survey included an error in the phrasing of item (5) and thus it was excluded from all analyses

	Relative frequency (*n* = 36)				
	Never	Rarely	Sometimes	Often	Always
1. In a paper, failing to report all of a study’s dependent measures	13	17	6		
2. Deciding whether to collect more data after looking to see whether the results were significant	20	11	4	1	
3. In a paper, failing to report all of a study’s conditions	18	17	1		
4. Stopping collecting data earlier than planned because one found the result that one had been looking for	26	9	1		
6. In a paper, selectively reporting studies that ‘worked’	19	14	2	1	
7. Deciding whether to exclude data after looking at the impact of doing so on the results	26	10			
8. In a paper, reporting an unexpected finding as having been predicted from the start	24	5	4	2	1
9. In a paper, claiming that results are unaffected by demographic variables when one is actually unsure	29	7		1	
10. Falsifying data	34	2			

### Procedure

Participants completed the study online. It was programmed in jsPsych (De Leeuw, 2015) and hosted on Pavlovia. Participants completed the study on their own computers.

#### The AX discrimination task

This task was completed in three blocks of 50 stimuli each, with each experimental stimulus and two matched control stimuli randomly assigned to different blocks, and the stimuli within each block presented in a random order for each participant. Half of the experimental stimuli and their matching control stimuli were selected to have the larger *p*-value presented first. On each trial, the first *p*-value was presented for 1000 ms. Next, the other *p*-value was presented just below it. Participants were instructed to “determine whether the two *p*-values are similar to or different from each other”. They pressed the ‘F’ key if they judged the *p*-values to be “similar” and the ‘J’ key if they judged them to be “different”. Participants were instructed to respond as quickly as possible but no time limit was enforced. Beforehand, they completed a short practice block of seven practice trials. Participants had the opportunity to rest between blocks.

#### The ABX discrimination task

This task was completed in four blocks of approximately 50 stimuli each, with each experimental triad and the two matched control triads randomly assigned to different blocks, and the stimuli within each block presented in a random order for each participant. On each trial, the end *p*-values in the triad were presented simultaneously, with the smaller one on the left and the larger on the right; this ordering is congruent with that of the MNL (Dehaene et al., 1993). After 750 ms, the middle *p*-value was then presented below and centered between the end *p*-values. Participants were instructed to judge whether the middle *p*-value was “more like” the left/smaller or right/larger *p*-value, pressing the ‘F’ and ‘J’ keys, respectively. Again, they were instructed to make their judgments as quickly as possible but no time limit was enforced. Beforehand, they completed a short practice block of seven practice trials. They had the opportunity to rest between blocks. The 102 asymmetric stimuli triads were presented randomly interspersed with 96 symmetric stimuli.

#### The QRP and participant survey

The graduate students also completed the QRP survey. They were asked to rate 10 different QRPs on “how often they are acceptable”, with response options of “never acceptable”, “rarely acceptable”, “sometimes acceptable”, “often acceptable”, and “always acceptable” (see Table 4). They then answered questions related to their major or area of research, their previous training in statistics, and their familiarity with *p*-values and the 0.05 threshold. Next, they were asked about possible demand characteristics of the study, specifically what they thought the tasks were meant to assess, and whether their guess about the study purpose affected their behavior. The survey also included items asking for demographic information such as age and field of study. Finally, it queried engagement with open science initiatives. See the Additional file 1 for the full survey.

## Results

### Study and trial completion times

The median total time to complete the study was 36 min for graduate students (IQR: 33–45) and 24 min for undergraduates (IQR: 20–29). (Recall that the undergraduates did not complete the filler task or the QRP survey.) The two groups took roughly equal amounts of time to perform each trial of the AX and ABX tasks, with a median trial response time (RT) of 884 ms for graduate students (IQR: 700–1130 ms) compared to 835 ms for undergraduates (IQR: 676–1067 ms).

### The AX task

Data from five graduate students and 10 undergraduate students were excluded from the analysis of the AX task for failure to comply with instructions as evidenced by their response profiles (e.g., 100% of responses marked as “different” or an average RT less than 150 ms per trial, which is well below the minimum required for processing numbers). Thus, the data analyzed for this task consisted of 37 graduate students and 39 undergraduates.

Following Rao et al. (2022), participants’ responses were analyzed with mixed-effects log-binomial models with a selection of “different” as the outcome. Random effects included whether the stimulus pair crossed 0.05 and a random intercept for between-participant differences in the subjective interpretation of “similar” and “different”. Fixed effects included the distance between *p*-values in the pair, the average size of the *p*-values, and whether the first *p*-value presented was smaller than the second.

**Fig. 2:**
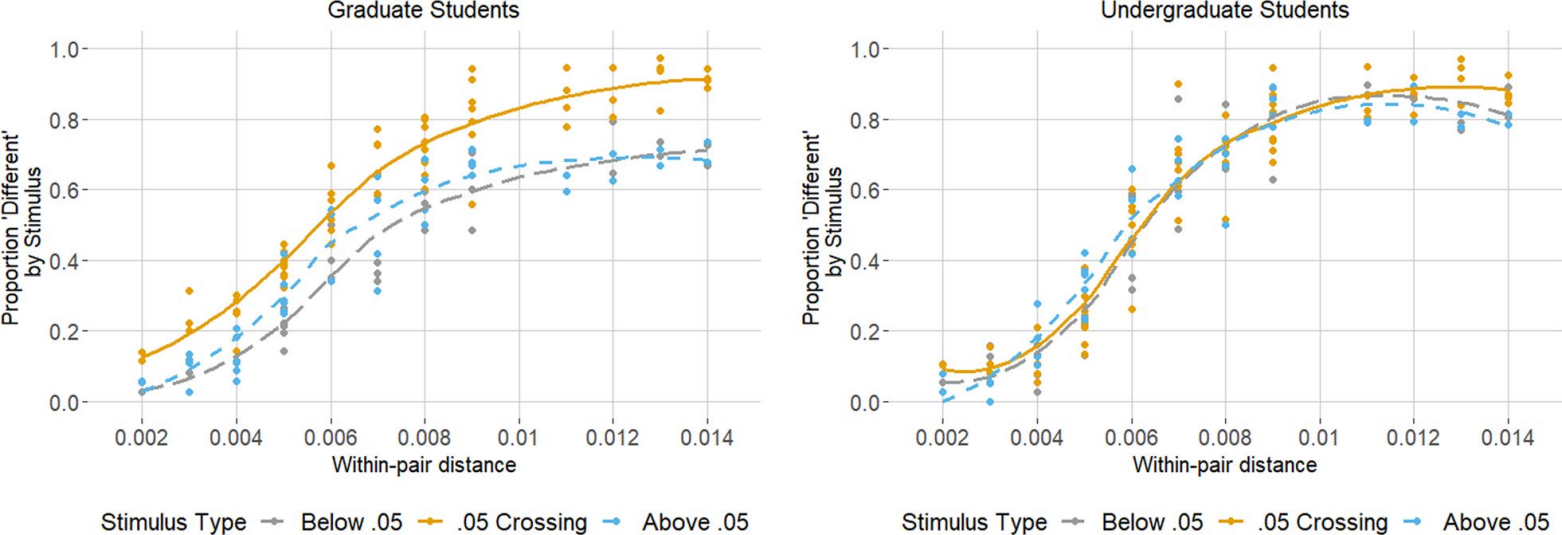
Unadjusted Results of the AX Discrimination Task for Graduate Students (left panel) and Undergraduate Students (right panel). On average, for each unique stimulus (each dot), graduate students but not undergraduate students were more likely to judge two p-values as different when they crossed the 0.05 boundary (solid orange line) than when they crossed a different hundredths boundary, i.e., one above (short-dashed blue line) or below (long-dashed grey line). This was true regardless of the absolute distance between the pair

Graduate students were an estimated 1.805 (95% CI [1.29, 2.52], *p* < 0.001) times as likely (i.e., 80.5% more likely) to judge a pair of *p*-values as “different” when they crossed 0.05 versus when they did not; see the left panel of Fig. 2. Thus, the boundary effect observed previously in Rao et al. (2022) for the AX task replicated here in a larger sample. Model results also suggest a distance effect on graduate students’ selections (95% CI rate ratio for selecting “different” per 0.001 increase in within-pair distance [1.12, 1.15], *p* < 0.001) as expected, with graduate students more likely to judge two *p*-values as different as the distance between them increased. Graduate students’ selections were likely not predominantly affected by the size of the *p*-values (*p* = 0.100)—as predicted by the standard logarithmically-compressed MNL model—nor whether the smaller or larger *p*-value was presented first (*p* = 0.221). (For the full statistical output, see Additional file 1: Table S3).

Strikingly, model results for undergraduates suggest no clear boundary effect (as was the case with graduate students), as depicted in the right panel of Fig. 2. There was little if any difference in how frequently they judged *p*-values as “different” when they crossed 0.05 versus when both were on the same side (Rate Ratio = 1.069, 95% CI [0.987–1.16], *p* = 0.102). This suggests that 0.05 is not a domain-general landmark for rational numbers in this task; rather, the boundary effect observed among graduate students reflects a domain-specific demarcation due to statistical training about *p* < 0.05. Model results suggest a distance effect on undergraduates’ response selections (95% CI rate ratio for selecting “different” per 0.001 increase in within-pair distance [1.14, 1.17], *p* < 0.001) of similar magnitude as the estimated distance effect for graduate students. Undergraduates’ selections were likely not predominantly driven by the size of the *p*-values nor whether the smaller *p*-value was displayed first (*p*s > 0.70). (For the full statistical output, see Additional file 1: Table S4.)

**Fig. 3:**
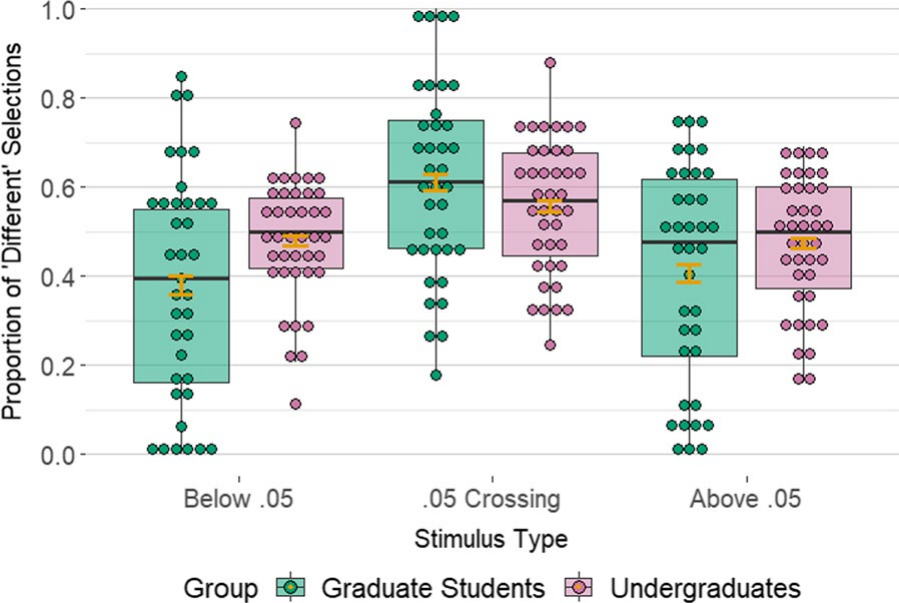
Unadjusted distribution of the proportion of ‘different’ selections across all AX stimuli for each participant, shown by group and stimulus type. Orange error bars represent 95% confidence intervals for the mean

CPE-like boundary effects manifest as both exaggerated cross-category differences and diminished within-category differences. Thus, we directly compared graduate students’ estimated boundary effects to undergraduate students for the 0.05 crossing stimuli and those stimuli where both *p*-values lie on the same side of 0.05 (i.e., the “Below 0.05” and “Above 0.05” stimuli), looking for an interaction; see Fig. 3. For stimuli crossing 0.05, graduate students were 1.325 times as likely (i.e., 32.5% more likely) to judge the *p*-values as “different” relative to undergraduates (95% CI [1.19–1.47], *p* < 0.001). Thus, they experienced an *exaggerated* psychological difference across 0.05. Conversely, graduate students were only 0.769 times as likely (i.e., 23.1% less likely) to judge *p*-values as “different” when both were on the same side of 0.05, relative to undergraduates (95% CI [0.68–0.87], *p* < 0.001). Thus, their psychological differences were *diminished* across boundaries other than 0.05. Together, the exaggerated cross-category difference and diminished within-category difference are strong evidence of a boundary effect. (Model results suggest no other differences in effects on response selections between the two groups.)

### The ABX task

Data from nine graduate students and 14 undergraduates were excluded from the analysis of the ABX task for the same reasons as in the AX task, leaving 33 graduate students and 34 undergraduates in the data analyzed for this task. As with the AX task, responses were analyzed with mixed-effects log-binomial models. Unlike the AX trials (and the symmetric filler ABX trials), on the asymmetric ABX trials of interest, there is an arithmetically correct response. However, the prompt (‘more like’) was meant to elicit intuitive and subjective judgments. We analyzed participants’ selections of the arithmetically incorrect responses, i.e., the selection of the *p*-value with the *same hundredths digit* as the outcome (e.g., for the triad {0.046, 0.049, 0.051}, selecting 0.046 as “more like” 0.049). If 0.05 is indeed a psychologically-real boundary for graduate students, then they should make this arithmetically incorrect selection at a higher rate when the *p*-values cross 0.05 than when they cross other hundredths-place boundaries. Specifically, this rate should be higher than for undergraduates because only graduate students experience a psychological boundary at 0.05.

**Fig. 4:**
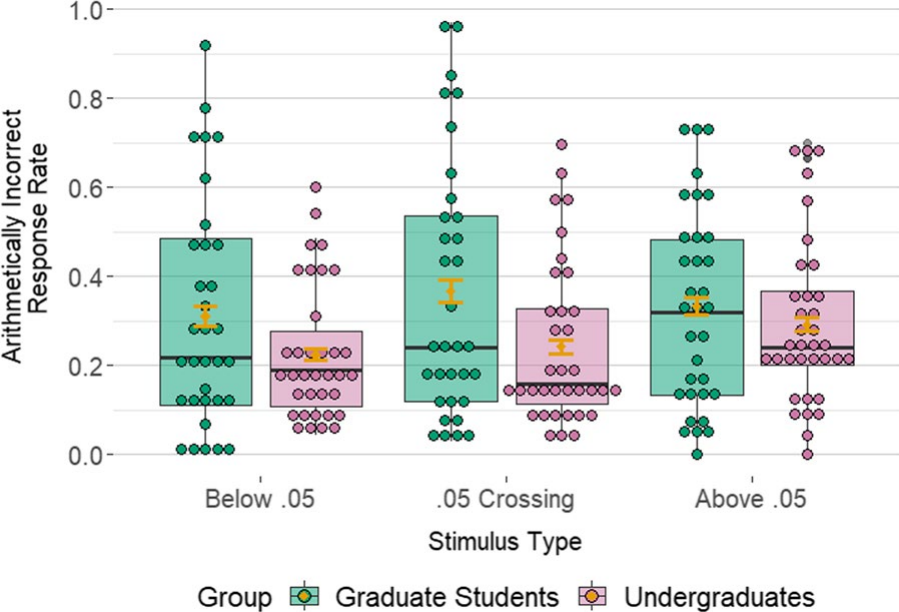
Unadjusted distribution of the proportion of ‘arithmetically incorrect’ selections across all ABX stimuli for each participant, shown by group and stimulus type. Orange error bars represent 95% confidence intervals for the mean

After adjusting for covariate effects of distance, decade-crossing, and size, graduate students were an estimated 1.135 times (95% CI [1.01, 1.28]; *p* = 0.038) as likely (i.e., 13.5% more likely) to select the same-hundredths-digit *p*-value (i.e., the arithmetically incorrect one) when the triad crossed 0.05 relative to when it crossed a different hundredths boundary; see Fig. 4 and Additional file 1: Table S5. This effect was in the expected direction, providing further evidence that 0.05 is a psychologically real boundary for graduate students. With respect to the covariates, model results suggest the distance between *p*-values also affected participants’ selections (*p* < 0.001): the further away the end *p*-value with the same hundredths digit (i.e., the arithmetically incorrect one) was from the middle *p*-value, the less likely participants were to select it. Results suggest that none of the other factors in the model were predominant factors affecting participants’ responses.

By comparison, model results suggest that undergraduates’ responses were not driven by a 0.05 boundary effect, as was the case with graduate students. They were an estimated 1.009 times (95% CI [0.91–1.11]; *p* = 0.865) as likely (i.e., 0.9% more likely) to say that the middle *p*-value was more like the *p*-value with the same hundredths digit (i.e., the arithmetically incorrect one) when the triad crossed 0.05 relative to when it crossed a different hundredths boundary (see Fig. 4 and Additional file 1: Table S6). With respect to the covariates, results suggest that undergraduates’ responses were affected by a distance effect similar to the one observed for graduate students (*p* < 0.001). They also likely were affected by a size effect: undergraduates were 1.296 times as likely (i.e., 29.6% more likely) to pick the same-hundredths-digit *p*-value (i.e., the arithmetically incorrect one) per 0.01 increase in the size of the *p*-values (95% CI [1.03, 1.63], *p* = 0.027). To summarize, the evidence suggests there was no special boundary effect at 0.05 for undergraduates: hundredths-place effects were found across the board.

Comparing graduate students to undergraduates, graduate students were 1.126 times as likely (i.e., 12.6% more likely) to say that the middle *p*-value was more like the same hundredths digit *p*-value (i.e., the arithmetically incorrect one) when the triad crossed 0.05 relative to undergraduates (95% CI: 0.97–1.31 times as likely, *p* = 0.064); see Fig. 4. Although the statistical evidence is not overwhelming in this case, the direction of the effect is consistent with the hypothesis that 0.05 is a psychological boundary for the graduate students, who have increased training on and exposure to it, more so than for undergraduates.

The undergraduates were more accurate in their responses than graduate students across all stimulus types. That this was the case for the control stimuli is counterintuitive at first glance. However, it is also consistent with the overall prediction. If graduate students have the category ‘Below 0.05’, then they should see the choice between *p*-values below 0.05 (e.g., is *p* = 0.032 more like *p* = 0.027 or *p* = 0.036?) as arbitrary because all three are the same in terms of their category membership. Thus, their judgments should be less driven by arithmetic distance, and they should be less accurate on the ‘Below 0.05’ stimuli. The same reasoning holds for ‘Above 0.05’ stimuli. By contrast, if undergraduates do not possess these categories, then they should be more driven by arithmetic distance, and thus more accurate on the control stimuli. This was indeed the case, lending further credence to the hypothesis that 0.05 is a psychological boundary for graduate students more so than for undergraduates.

### The QRP survey

Table 4 presents the response distributions of the graduate students to the QRP survey items. As in John et al. (2012), almost all participants indicated that “falsifying data” is never acceptable. The dominant pattern is that participants rated most practices as rarely or never acceptable. See Additional file 1: Table S7 for the average responses to the practices of our participants compared to those of John et al. (2012).

To evaluate whether graduate students with a larger estimated boundary effect tend to find QRPs more acceptable, we utilized log binomial mixed-effects models fitted separately to each of the participants’ responses for each of the AX and ABX tasks. This generated individual estimates of the 0.05 boundary effect for each of the tasks. We converted participants’ responses to survey questions to a numerical scale using a process similar to John et al. (2012), with “never acceptable” corresponding to 0, “rarely” to 1, “sometimes” to 2, “often” to 3, and “always” to 4. We then computed acceptability scores for each participant as the average of their responses across the 9 items.

**Fig. 5:**
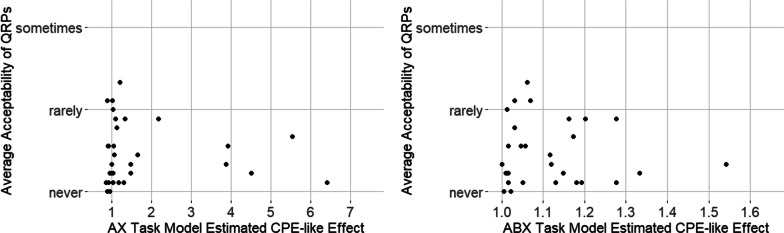
Relationship between the model-estimated 0.05 boundary effects from the AX task (left panel) and ABX task (right panel) with the average acceptability rating (0 = never; 1 = rarely; 2 = sometimes) across the nine QRP items, for each graduate student participant

There was no evidence of a meaningful association between the size of participants’ boundary effects and their QRP responses. This is shown in the left panel of Fig. 5 for the AX task (Kendall’s *τ* = 0.09, *p* = 0.481) and in the right panel for the ABX task (Kendall’s *τ* = 0.08; *p* = 0.517). Thus, there was no support for the proposal that the moment-to-moment cognitive processing of graduate students is linked to their explicit self-reported beliefs about the acceptability of QRPs.

## Discussion

This study moves the debate about *p*-values and NHST from meta-science to psychological science. We investigated whether through statistical training and reading the Results sections of a literature still dominated by NHST, 0.05 transcends from just a matter of statistical convention to become a psychological boundary in the minds of emerging scientists. The question of the psychological status of statistical recommendations is not entirely new (e.g., Bishop, 2020; Rosenthal & Gaito, 1963). Here, we built on our recent work (Rao et al., 2022) using the tools of categorical perception to shed new light on this question.

We first used the AX paradigm to replicate previous findings (Rao et al., 2022) that emerging scientists are more likely to judge *p*-values as different when they cross 0.05 compared to when they do not. Going beyond prior work, we also found that undergraduates did not show the same sensitivity to the 0.05 boundary.

We then extended this psychological boundary effect for the first time to the ABX paradigm, a stronger test of CPE-like effects (e.g., Greenaway, 2017). Participants saw triads of ordered *p*-values (e.g., {0.045, 0.051, 0.059}) and had to judge which end value the middle value was “more like”. When 0.05 was between the middle value and an arithmetically closer end value (here, 0.045 and 0.051), emerging scientists were more likely to choose the other end value—the one on the same side of 0.05 (here, 0.059)—even though it was arithmetically more distant. Again, the undergraduates did not show this same sensitivity to the 0.05 boundary.

Collectively, the findings from the AX and ABX tasks suggest that emerging scientists’ CPE-like boundary effects for *p*-values are a domain-specific consequence of their statistical training in NHST and their reading of a literature in which “*p* < 0.05” categorically demarcates “statistically significant” findings from null results.

One challenge to this conclusion is that these findings might instead reflect pre-existing differences in the numerical processing of emerging scientists compared to undergraduates, and thus are the result of selection biases. This could be assessed in a future longitudinal study evaluating whether sensitivity to the 0.05 boundary emerges during graduate training and increases further across post-graduate life in science (Nelson et al., 1986).

Another challenge is that the emerging scientists may have detected the purpose of the study and responded in a manner they thought we desired. In fact, this does not appear to have been the case. An open-ended item in the post-experiment survey asked participants what they thought the purpose of the study was. Of the 42 emerging scientists, 24 correctly inferred that the research goal of the AX task was to measure whether *p-*value pairs crossing 0.05 are perceived as less similar than pairs below or above 0.05. We repeated the analysis above for the 18 remaining emerging scientists who did *not* guess the specific research goal. Their results still appear to be governed by a boundary effect: They were an estimated 1.544 (95% CI [1.06, 2.25], *p* = 0.024) times as likely to judge a pair of *p*-values as “different” when they crossed 0.05 versus when they did not. (See Additional file 1: Table S8 for the full statistical output.) A parallel analysis of the ABX task found that the 18 graduate students who did not guess the specific research goal of the study were an estimated 1.156 times (95% CI [0.97, 1.37]; *p* = 0.098) more likely to select the same-hundredths-digit *p*-value (i.e., the arithmetically incorrect one) when the triad crossed 0.05 relative to when it crossed a different hundredths boundary. (See Additional file 1: Table S9 for the full statistical output).

Speeded processing of *p*-values occurs on a quite different timeline than the production of scientific knowledge. For this reason, we adapted the John et al. (2012) QRP survey to assess whether psychological boundary effects at 0.05 as measured in the AX and ABX tasks are associated with QRP acceptability ratings. We predicted that there would be a positive association between the size of the 0.05 boundary in emerging scientists and how acceptable they find QRPs. This prediction found no empirical support.

There are at least three interesting explanations for this lack of association, beyond the usual suspects (i.e., insufficient sample size, measurement reliability, response bias due to social desirability effects). The first is that over the past 10–15 years, psychological science has broadly acknowledged the replication crisis and the NHST controversy. Thus, new students might be enculturated into a different set of beliefs, values, and practices than more experienced researchers. Consistent with this, our participants showed a floor effect, with almost all responses to almost all QRPS either “never” or “rarely”. (Note that floor effects inhibit the ability to detect differences between groups or relationships between factors, which might also explain the lack of association.) By contrast, there was no such floor effect among faculty participants 10 years ago (John et al., 2012). The second explanation is that our participants lacked sufficient practical research experience compared to the faculty at PhD-granting universities in John et al. (2012). Emerging scientists may endorse a stricter interpretation of methodological maxims that does not reflect the pragmatic challenges of working scientists.

The third explanation is perhaps the most interesting. Science occurs at multiple timescales. Oversensitivity to the 0.05 boundary might be a feature of scientists’ moment-by-moment thinking. The same might be said of confirmation bias and other deviations from normative reasoning (Bishop, 2020). Over longer timescales, however, the processes of science might work to mitigate these deviations. Thus, when explicitly asked about QRPs from their own labs, scientists might judge them to be unacceptable, and they might mean it.

Note that both the AX and ABX tasks are speeded judgments. We do not propose that researchers’ ability to perform explicit decimal subtraction is irrevocably damaged in the *p*-value context, merely that CPE-like boundary effects play a role in *p*-value processing. Thus, it is important to examine a potential link between the in-the-moment cognitive processing of individual psychological scientists and the broader replication crisis in psychological science.

We close with a word of caution to those who have long since dismissed “*p* < 0.05” from their thinking about data: There is no reason to believe that the boundary effects observed here are limited to *p*-values or the NHST framework. Moving to a new framework that also includes *magic numbers*—especially dichotomies—will likely bring versions of the boundary effects we have documented. This is because categorization is a general feature of human cognition (Goldstone, 2003; Harnad, 2017). Categories are formed when people learn to demarcate continua, regardless of the statistical school to which they belong. We likewise predict that pairs of Bayes factors that cross conventional boundaries (e.g., 3 or 1/3) will be perceived as psychologically more different than those that fall on the same side. While statistical guidelines may be useful, their entrenchment may also carry psychological side effects. 

### Supplementary Information

**Additional file 1**. contains supplementary tables of results.

## Data Availability

Anonymized copies of the data and materials for this study are publicly available at https://osf.io/jfkrp/. Relevant details of the materials are included in the manuscript and Additional file 1. Additionally, we report a power analysis to determine sample size, all data exclusions, all manipulations, and all measures in the study, and we follow JARS (Kazak, 2018). Data were analyzed using R, version 4.0.0 (R Core Team, 2020) and the package lme4, version 1.1–27.1 (Bates et al., 2015). This study’s design and analysis were not pre-registered.

## Abbreviations

CPE: Categorical perception effect
MNL: Mental number line
NHST: Null hypothesis significance testing
QRP: Questionable research practice

## Footnotes

1 This psychological categorization of *p*-values involves both perception and cognition of symbolic numbers in a specific context. We invoke CPEs as a framework, not as a literal claim about statistical cognition.

2 Item (5) of the John et al. (2012) survey is: "In a paper, ‘rounding off’ a *p* value (e.g., reporting that a *p* value of .054 is less than .05)”. Our implementation of the survey accidentally changed the parenthetical phrase to: “(e.g., reporting *p* = .054 as *p* = .05)”. This changes the meaning of the item enough that we elected to exclude it from all analyses.
