# Supplementary material for: The psychological reality of the learned “p < .05” boundary
Source: Cogn Res Princ Implic. 2024 May 3;9:27. doi: 10.1186/s41235-024-00553-x (PMC11068716; doi:10.1186/s41235-024-00553-x)
Supplement: Supplementary file 1 — Additional file 1. contains supplementary tables of results. [file 41235_2024_553_MOESM1_ESM.docx]

*Supplementary Materials for*

The Psychological Reality of the Learned “*p* < .05” Boundary

V.N. Vimal Rao^1^, Jeffrey K. Bye^1^, and Sashank Varma^2^

^1^ Department of Educational Psychology, University of Minnesota

^2^ School of Interactive Computing, School of Psychology, Georgia Institute of Technology

**Table S1**

*Number of Graduate Student Participants by Area of Specialization*

| Area of Specialization | Number |
| --- | --- |
| Clinical Psychology | 7 |
| Cognitive Psychology | 7 |
| Developmental/Child Psychology | 9 |
| Educational Psychology | 7 |
| Neuroscience | 2 |
| Personality Psychology | 1 |
| Measurement and Statistics | 5 |
| Social Psychology | 4 |

**Table S2**

*Number of Undergraduate Student Participants by Major Discipline*

| Major Discipline | Number |  |
| --- | --- | --- |
| Physical Sciences | 7 | |
| Psychological Sciences | 5 | |
| Social Sciences | 8 | |
| Computer Sciences | 4 | |
| Engineering | 9 | |
| Fine Arts | 2 | |
| Education | 3 | |
| Health Sciences | 5 | |
| Business | 3 | |
| Design and Architecture | 3 | |

**Table S3**

*Estimated Fixed Effects from the Fitted Log-Binomial Mixed Effects Model for Graduate students’ difference selections in the AX task*

| Factor |  | Difference selections | |
| --- | --- | --- | --- |
|  |  | Estimated rate-ratio (95% CI) | *p* |
| .05 Crossing |  | 1.805 (1.29 – 2.52) | < 0.001 |
| Distance* |  | 1.135 (1.12 – 1.15) | < 0.001 |
| Size* |  | 1.000 (0.99 – 1.00) | 0.100 |
| Smaller 1^st^ |  | 0.953 (0.88 – 1.03) | 0.221 |

*Estimates are per 0.001 increase

**Table S4**

*Estimated Fixed Effects from the Fitted Log-Binomial Mixed Effects Model for Undergraduate students’ difference selections in the AX task*

| Factor |  | Difference Selections | |
| --- | --- | --- | --- |
|  |  | Estimated rate-ratio (95% CI) | *p* |
| .05 Crossing |  | 1.069 (0.98 – 1.16) | 0.102 |
| Distance* |  | 1.156 (1.14 – 1.17) | < 0.001 |
| Size* |  | 1.000 (0.99 – 1.00) | 0.719 |
| Smaller 1^st^ |  | 1.014 (0.94 – 1.09) | 0.706 |

*Estimates are per 0.001 increase

**Table S5**

*Estimated Fixed Effects from the Fitted Log-Binomial Mixed Effects Model for Graduate students’ same-hundredths-digit selections in the ABX task*

| Factor |  | Hundredths-Crossing stimuli selections | |
| --- | --- | --- | --- |
|  |  | Estimated rate-ratio (95% CI) | *p* |
| .05 Crossing |  | 1.135 (1.01 – 1.28) | 0.038 |
| Hundredths-crossing Distance* |  | 1.071 (1.03 – 1.12) | < 0.001 |
| Same-Hundredths Distance* |  | 0.945 (0.91 – 0.98) | 0.004 |
| Size** |  | 1.160 (0.91 – 1.48) | 0.237 |
| Hundredths-Boundary between smallest and middle *p*-value |  | 0.939 (0.86 – 1.03) | 0.179 |

*Estimates are per 0.001 increase

**Estimates are per 0.010 increase

**Table S6**

*Estimated Fixed Effects from the Fitted Log-Binomial Mixed Effects Model for Undergraduate students’ same-hundredths-digit selections in the ABX task*

| Factor |  | Hundredths-Crossing stimuli selections | |
| --- | --- | --- | --- |
|  |  | Estimated rate-ratio (95% CI) | *p* |
| .05 Crossing |  | 1.009 (0.91 – 1.11) | 0.865 |
| Hundredths-crossing Distance* |  | 1.087 (1.05 – 1.13) | < 0.001 |
| Same-Hundredths Distance* |  | 0.937 (0.90 – 0.97) | < 0.001 |
| Size** |  | 1.296 (1.03 – 1.63) | 0.027 |
| Hundredths-Boundary between smallest and middle *p*-value |  | 0.990 (0.91 – 1.08) | 0.808 |

*Estimates are per 0.001 increase

**Estimates are per 0.010 increase

**Table S7**

*Comparison of QRP survey responses of Graduate Students to John et al. (2012); our implementation of the survey included an error in the phrasing of item (5) and thus it was excluded from all analyses*

| Item |  | Participant Group | |
| --- | --- | --- | --- |
|  |  | Graduate Students* | John et al. (2012)** |
| 1. In a paper, failing to report all of a study’s dependent measures |  | 0.83 | 1.84 |
| 2. Deciding whether to collect more data after looking to see whether the results were significant |  | 0.64 | 1.79 |
| 3. In a paper, failing to report all of a study’s conditions |  | 0.55 | 1.77 |
| 4. Stopping collecting data earlier than planned because one found the result that one had been looking for |  | 0.36 | 1.76 |
| 6. In a paper, selectively reporting studies that ‘worked’ |  | 0.64 | 1.66 |
| 7. Deciding whether to exclude data after looking at the impact of doing so on the results |  | 0.36 | 1.61 |
| 8. In a paper, reporting an unexpected finding as having been predicted from the start |  | 0.74 | 1.50 |
| 9. In a paper, claiming that results are unaffected by demographic variables when one is actually unsure |  | 0.36 | 1.32 |
| 10. Falsifying data |  | 0.05 | 0.16 |

*Average Acceptability ratings measured 0=“never”, 1=“rarely”, 2=“sometimes”, 3=“often”, 4=“always”

**Average Defensibility ratings measured 0=“no”, 1=“possibly”, or 2=“yes”

**Table S8**

*Estimated Fixed Effects from the Fitted Log-Binomial Mixed Effects Model for Graduate students’ difference selections in the AX task for only those Graduate students who did not guess the purpose of the study*

| Factor |  | Difference selections | |
| --- | --- | --- | --- |
|  |  | Estimated rate-ratio (95% CI) | *p* |
| .05 Crossing |  | 1.544 (1.06 – 2.25) | 0.024 |
| Distance* |  | 1.141 (1.12 – 1.16) | < 0.001 |
| Size* |  | 1.000 (0.99 – 1.00) | 0.932 |
| Smaller 1^st^ |  | 0.969 (0.88 – 1.07) | 0.528 |

*Estimates are per 0.001 increase

**Table S9**

*Estimated Fixed Effects from the Fitted Log-Binomial Mixed Effects Model for Graduate students’ same-hundredths-digit selections in the ABX task for only those Graduate students who did not guess the purpose of the study*

| Factor |  | Hundredths-Crossing stimuli selections | |
| --- | --- | --- | --- |
|  |  | Estimated rate-ratio (95% CI) | *p* |
| .05 Crossing |  | 1.156 (0.97 – 1.37) | 0.098 |
| Hundredths-crossing Distance* |  | 1.081 (1.03 – 1.14) | 0.004 |
| Same-Hundredths Distance* |  | 0.935 (0.89 – 0.98) | 0.009 |
| Size** |  | 1.116 (0.81 – 1.54) | 0.503 |
| Hundredths-Boundary between smallest and middle *p*-value |  | 0.983 (0.87 – 1.11) | 0.775 |

*Estimates are per 0.001 increase

**Estimates are per 0.010 increase

**Full QRP and Participant Survey**

You have now finished all tasks.

The final part of this study involves a survey.

Please rate the following practices on how often they are acceptable.

[never acceptable, rarely acceptable, sometimes acceptable, often acceptable, always acceptable]

1. In a paper, failing to report all of a study's dependent measures
2. Deciding whether to collect more data after looking to see whether the results were significant
3. In a paper, failing to report all of a study's conditions
4. Stopping collecting data earlier than planned because one found the result that one had been looking for
5. In a paper, 'rounding off' a p-value (e.g., reporting p = 0.054 as p=0.05)
6. In a paper, selectively reporting studies that 'worked'
7. Deciding whether to exclude data after looking at the impact of doing so on the results
8. In a paper, reporting an unexpected finding as having been predicted from the start
9. In a paper, claiming that results are unaffected by demographic variables when one is actually unsure
10. Falsifying data

What is your age group?

['18 to 24', '25 to 30', '31 to 40', '41 to 50', '51 to 60', '61 and over', 'Prefer not to say']

Which of the following best describes your gender identity?

['Woman', 'Man', 'Non-binary', 'Prefer to self-describe', 'Prefer not to say']

Which of the following best describes your ethnicity or race? Select all that apply to you.

[‘Alaska Native or American Indian - For example, Nome Eskimo Community, Native Village of Barrow Inupiat Traditional Government, Blackfeet Tribe, Mayan, Aztec, Navajo Nation, etc.’,

‘Asian - For example, Chinese, Filipino, Asian Indian, Vietnamese, Korean, Japanese’,

‘Black or African American - For example, Jamaican, Haitian, Nigerian, Ethiopian, Somali’,

‘Hispanic, Latino or Spanish Origin - For example, Mexican or Mexican American, Puerto Rican, Cuban, Salvadoran, Dominican, Columbian’,

‘Middle Eastern or North African - For example, Lebanese, Iranian, Egyptian, Syrian, Moroccan, Algerian’,

‘Pacific Islander - For example, Native Hawaiian, Samoan, Chamorro, Tongan, Fijian, Marshallese’,

‘White - For example, of German, Irish, English, Italian, Polish, French descent’,

‘Some other race or ethnicity, please specify (next page)’,

‘Prefer not to say’]

Please specify your area(s) of research (e.g., cognitive neuroscience).

How familiar are you with *p*-values?

['not familiar at all', 'somewhat familiar', 'very familiar']

How familiar are you with the .05 threshold associated with *p*-values?

['not familiar at all', 'somewhat familiar', 'very familiar']

When did you enter graduate school?

['Prior to 1980', '1980 to 1989', '1990 to 1999', '2000 to 2005', '2006 to 2010', '2011 to 2015', '2016 to 2021', 'Prefer not to say']

When did you finish graduate school?

['Prior to 1980', '1980 to 1989', '1990 to 1999', '2000 to 2005', '2006 to 2010', '2011 to 2015', '2016 to 2021', 'Have not finished yet', 'Prefer not to say']

What best describes your current position?

['Undergraduate student', 'Graduate student', 'Postdoctoral researcher', 'Research associate', 'Lecturer', 'Assistant Professor', 'Associate Professor', 'Professor', 'Some other position, please specify (next page)', 'Prefer not to say']

Please indicate which of the following 'open science' activities you have participated in before:

[‘Participate in journal clubs discussing open science (e.g., ReproducibiliTea)’,

‘Attend or participate in a meta-science conference (e.g., SIPS)’,

‘Pre-register a study design/analysis’, ‘Submit a Registered Report’,

‘Publicly share study materials (open materials)’, ‘Publicly share data (open data)’,

‘Publicly share reproducible code for data analysis’, ‘Publicly share null results (e.g., in a preprint)’,

‘Share a preprint’, ‘Submit a signed review’, ‘Publish a paper in an open access journal’,

‘Serve as an editor/reviewer for an open access journal’,

‘Some other open science activities, please specify (next page)’,

 ’Prefer not to say’]

In the first task, you saw two *p*-values and made a judgment of whether the values were 'similar' or 'different'. What do you think this task was meant to assess?

Assuming your guess on the previous question is correct, do you believe figuring out the purpose of the task affected your behavior?  If so, how?

If a paper describes a result as 'statistically significant', what is the first *p*-value that you think of?

If a paper describes a result as 'highly statistically significant', what is the first *p*-value that you think of?

If a paper describes a result as 'marginally statistically significant', what is the first *p*-value that you think of?

What are the upper and lower bounds (range) of Cohen's *d* for what you would consider a small effect size?

What are the upper and lower bounds (range) of Cohen's *d* for what you would consider a medium effect size?

What are the upper and lower bounds (range) of Cohen's *d* for what you would consider a large effect size?

How familiar are you with Cohen's *d* effect sizes?

['not familiar at all', 'somewhat familiar', 'very familiar']

How familiar are you with the .2/.5/.8 guidelines for Cohen's *d* effect sizes?

['not familiar at all', 'somewhat familiar', 'very familiar']

We should use Bayesian statistical analyses (instead of frequentist) more often in psychology.

['Strongly disagree', 'Disagree', 'Agree', 'Strongly agree', ‘I'm not sure’]

You're done! Thank you for completing the study.
